# The essential role of TAZ in normal tissue homeostasis

**DOI:** 10.1007/s12272-021-01322-w

**Published:** 2021-03-26

**Authors:** Mi Gyeong Jeong, Hyo Kyeong Kim, Eun Sook Hwang

**Affiliations:** College of Pharmacy and Graduate School of Pharmaceutical Sciences, Ewha Womans University, C206 Science building, 52 Ewhayeodae-Gil, Seodaemun-Gu, Seoul, 03760 Korea

**Keywords:** Hippo pathway, TAZ, Tissue homeostasis, Transcriptional control, YAP

## Abstract

Transcriptional coactivator with PDZ-binding motif (TAZ) has been extensively characterized in organ development, tissue regeneration, and tumor progression. In particular, TAZ functions as a Hippo mediator that regulates organ size, tumor growth and migration. It is highly expressed in various types of human cancer, and has been reported to be associated with tumor metastasis and poor outcomes in cancer patients, suggesting that TAZ is an oncogenic regulator. Yes-associated protein (YAP) has 60% similarity in amino acid sequence to TAZ and plays redundant roles with TAZ in the regulation of cell proliferation and migration of cancer cells. Therefore, TAZ and YAP, which are encoded by paralogous genes, are referred to as TAZ/YAP and are suggested to be functionally equivalent. Despite its similarity to YAP, TAZ can be clearly distinguished from YAP based on its genetic, structural, and functional aspects. In addition, targeting superabundant TAZ can be a promising therapeutic strategy for cancer treatment; however, persistent TAZ inactivation may cause failure of tissue homeostatic control. This review focuses primarily on TAZ, not YAP, discusses its structural features and physiological functions in the regulation of tissue homeostasis, and provides new insights into the drug development targeting TAZ to control reproductive and musculoskeletal disorders.

## Introduction

Transcriptional coactivator with PDZ-binding motif (TAZ) has been identified as a phosphoprotein that interacts with 14–3-3 protein and its activity is controlled by nuclear-cytosol localization in a phosphorylation-dependent manner (Kanai et al. 2000). TAZ shares structural and functional similarities with yes-associated protein (YAP) (Macias et al. 1996; Piccolo et al. 2014). TAZ and YAP are phosphorylated and trapped in the cytoplasm via interaction with 14–3-3 or undergo proteasomal degradation through β-TrCP-mediated ubiquitination in response to Hippo signaling (Tian et al. 2007; Liu et al. 2010). The upstream mammalian Ste20-like kinases 1 and 2 (MST1/2) trigger the phosphorylation and activation of large tumor suppressor kinases 1 and 2 (LATS1/2) in the Hippo signaling cascade, which in turn phosphorylate TAZ/YAP and control their cytoplasmic retention and protein stability (Pan 2010; Yu and Guan 2013; Piccolo et al. 2014). When the Hippo signaling pathway is inactive, TAZ/YAP are dephosphorylated and localized in the nucleus. TAZ and YAP are associated with many transcription factors that control various physiological cellular events, including cell proliferation, differentiation, migration, apoptosis, and senescence, and thereby result in transcriptional activation of target genes (Varelas et al. 2008; Di Palma et al. 2009; Jeong et al. 2010, 2017; Wang et al. 2016; Kim et al. 2019, 2020). In particular, TAZ and YAP interact with transcription factors, in particular, transcriptional enhanced associate domain (TEAD) family and enhance the transcription of multiple targets involved in tumorigenesis, thereby affecting the self-renewal of stem cells, tumor progression, metastasis, and drug resistance (Chan et al. 2009; Zanconato et al. 2015, 2016; Lin et al. 2017). Nuclear localization and activation of TAZ and YAP are also regulated by mechanical signals, such as shear stress, cell shape, and extracellular rigidity (Dupont et al. 2011; Dupont 2016; Pocaterra et al. 2020). Although TAZ and YAP are paralogs with functional redundancy and tumor-promoting effects (Kim 2019), it is clear that TAZ and YAP play distinctively essential roles in normal tissue development and homeostasis (Yu et al. 2015; Reggiani et al. 2020). We clarify the genetic and protein structural features of TAZ and further highlight its biological functions in normal tissue differentiation from stem cells and in the regulation of tissue homeostasis.

### Structural features of TAZ

**Fig. 1:**
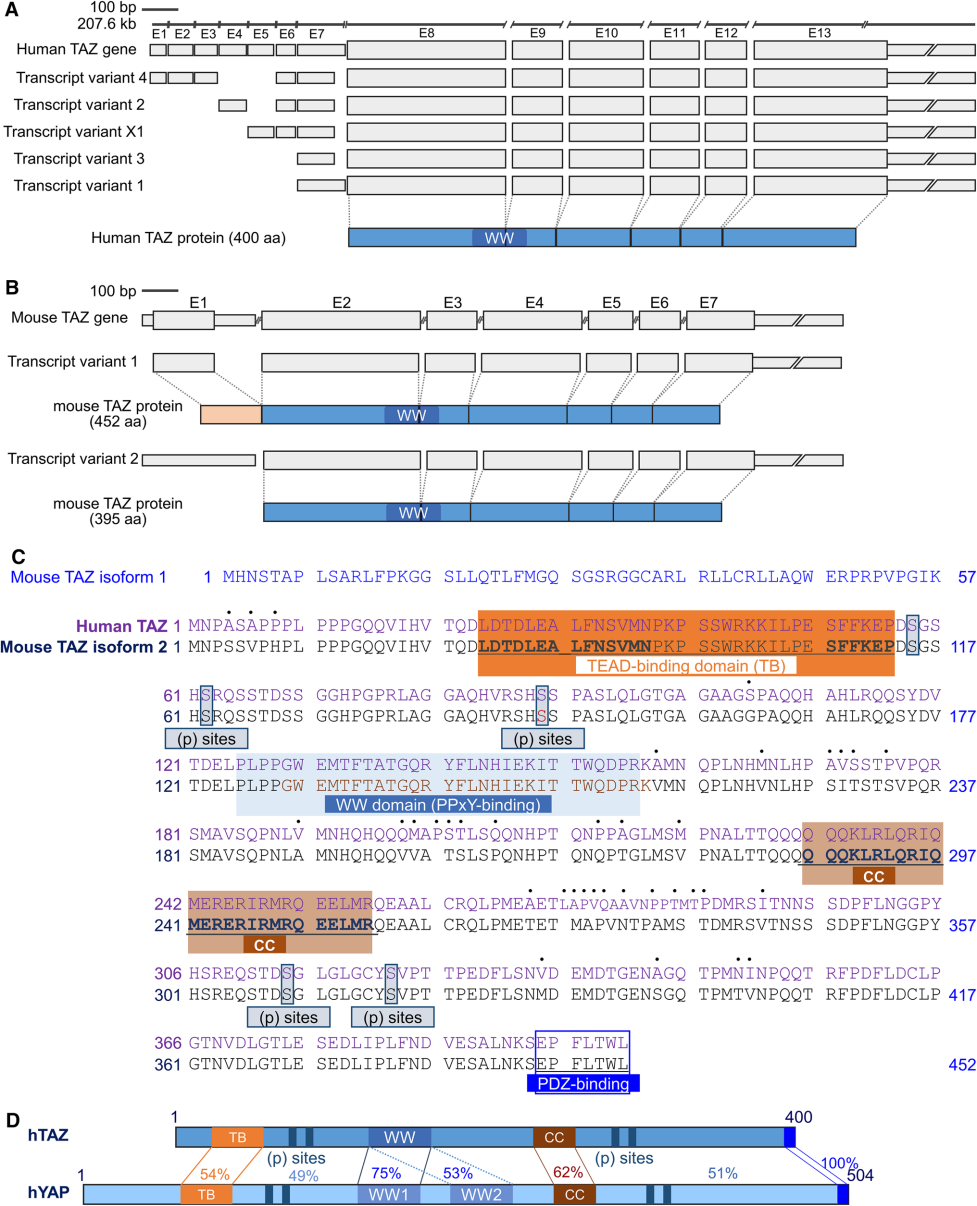
Structural features of TAZ. **a** Genome structure of human TAZ. Five different transcript variants of TAZ (NM_015472.6, NM_001168278.3, NM_001168280.3, NM_001348362.2, and XM_017006122.1) are produced from human chromosome 3 (GRCh38.p13) and are encoded as the same human TAZ comprising of 400 amino acids. **b** Mouse chromosome 3 (GRCm39) comprises of TAZ exons and introns. Two different transcript variants (NM_00168281.1 and NM_00168281.1) are generated and encoded as two isoforms of TAZ; isoform 1, 452 aa and isoform 2, 395 aa. **c** Amino acid alignment between human and mouse TAZ. Mouse TAZ isoform 1 contains additional 57 aa at the N-terminus and has no similarity with human TAZ. **d** Schematic structure of human and mouse TAZ proteins. The percent identity for a given sequence is presented. TB, TEAD-binding domain; WW, W-containing domain; CC, coiled-coil domain; and (p) sites, phosphorylation sites

*TAZ* gene is officially named as *WW domain-containing transcriptional coregulatory 1 (WWTR1)* gene, but it is more extensively and commonly used than *WWTR1*, and is different from *tafazzin (TAZ)*. The TAZ gene, a Hippo mediator, is highly conserved among divergent species, and the human TAZ gene has orthologs in 288 organisms, including zebrafish, frog, mouse, rat, chicken, cow, dog, pig, and chimpanzee. The 207.6 kb portion of the human TAZ gene located on chromosome 3 contains 13 exons and produces four different TAZ transcript variants, which encode the same 400 amino acid (aa) protein (Fig. 1a). In mouse, 7 exons transcribe two different transcript variants encoding two TAZ isoforms composed of 452 and 395 amino acids. The mouse TAZ isoform 1 is 100% homologous to mouse TAZ isoform 2 and additionally contains 57 aa at the N-terminal, which is not found in human TAZ. The human TAZ protein shares 91% identity with the mouse TAZ in the entire amino acid sequence, and the WW domain is completely identical (Fig. 1b). The WW domain is a modular protein domain composed of 40 aa, which mediates specific interactions with proteins containing P-rich motifs such as PPxY, LPxY, phosphor-(S/T)P, and PRR motifs (Hu et al. 2004). Many transcription factors, including Runt-related transcription factors (RUNXs), peroxisome proliferator-activated receptor γ (PPARγ), TEADs, SMADs, ErbB-4, and p73 are known to interact with the WW domain in TAZ or YAP (Strano et al. 2001; Ferrigno et al. 2002; Komuro et al. 2003; Zhao et al. 2009). Since TAZ contains a single WW domain unlike two WW domains in YAP, its interaction with TEAD is structurally different from TEAD-YAP (Kaan et al. 2017; Reggiani et al. 2020). TAZ can be distinguished from YAP by its interactions with many P-rich proteins and functions within the cells. In addition, the coiled-coil domain and PDZ-binding motif are within a larger transcriptional regulatory regions at the C-terminal domain of TAZ and serve as additional protein–protein interaction domains (Kanai et al. 2000; Hong et al. 2005).

### TAZ is essential for self-renewal and survival of stem cells

**Fig. 2:**
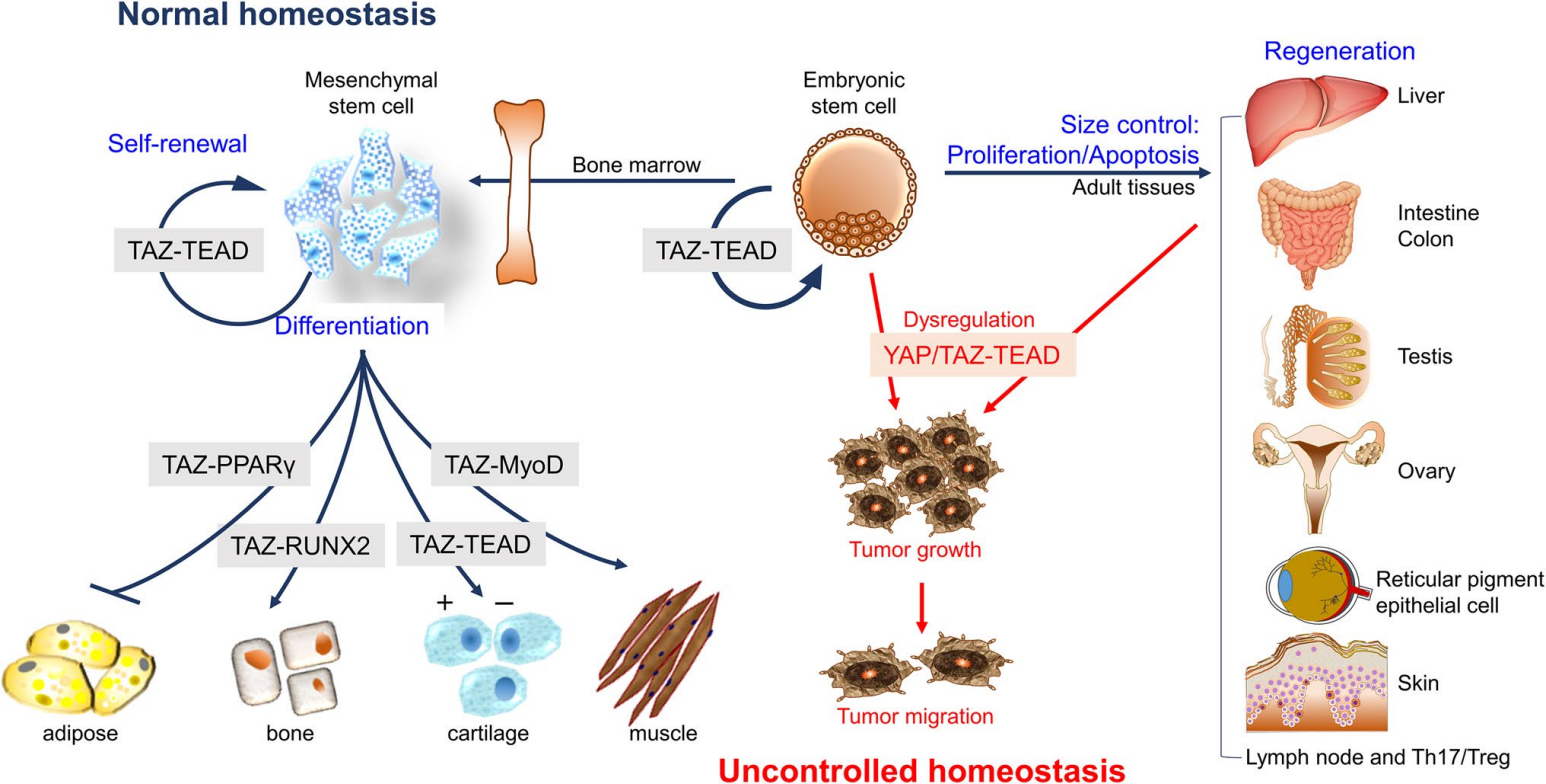
Control of tissue homeostasis by TAZ. Under normal homeostasis conditions, TAZ functions in the self-renewal of embryonic and mesenchymal stem cells through TAZ-TEAD complex formation. Differentiation of mesenchymal stem cells into different lineages is controlled by the association of TAZ with several transcription factors. Development of adult tissues and injury-induced tissue regeneration requires TAZ expression, which regulates organ size by controlling both cell proliferation and apoptosis. However, uncontrolled homeostatic imbalance may lead to irreversible changes in cells and tissues and subsequently to malignant tumor development. Overexpression or hyperactivation of YAP and TAZ accelerates tumor growth and metastasis by inducing dysregulation in cell proliferation, migration, and survival of stem cells

Stem cells are defined as undifferentiated cells capable of producing certain specialized cells and self-renewing progeny cells, and are classified as fetal, embryonic, and adult stem cells. TAZ is required to maintain the self-renewal and pluripotency of embryonic stem cells, since its deficiency in human embryonic stem cells leads to differentiation into neuroectodermal lineage (Varelas et al. 2008). TAZ also plays key roles in the expansion, self-renewal, and maintenance of stemness of tissue-specific adult stem cells. TAZ associates with snail/slug and cooperatively controls the self-renewal and osteoblastogenesis of adult skeletal stem cells (Tang et al. 2016; Tang and Weiss 2017). Exogenous TAZ expression reprograms primary differentiated mouse cells into tissue-specific stem cell or progenitor cell state, indicating the importance of TAZ for maintaining stemness of adult stem cells (Panciera et al. 2016). In addition, dysregulated TAZ expression in stem cells causes uncontrolled self-renewal and cell expansion, resulting in cancer growth and sustained survival of cancer stem cells (Bartucci et al. 2015; Mohamed et al. 2016; Elaimy et al. 2018). TAZ is crucial for maintaining stem cell population and the potency of both embryonic and adult stem cells (Fig. 2).

### TAZ modulates mesenchymal stem cell differentiation into bone, adipose, and muscle

Many transcription factors that contain P-rich motifs have been reported to interact with TAZ during the execution of many developmental programs (Hong and Yaffe 2006). TAZ associates with RUNX2 and strongly activates RUNX2-driven gene transcription, resulting in enhanced osteoblast differentiation (Hong et al. 2005; Long 2011). TAZ also interacts with PPARγ but markedly suppresses PPARγ-driven adipogenic and lipogenic gene expression, whereas TAZ deletion in mesenchymal stem cells increasingly drives adipocyte differentiation (Hong et al. 2005). TAZ is thus important for reprogramming of stem cell lineage commitment. While exogenous TAZ overexpression increased bone mineral density in vivo, TAZ deletion impaired osteogenic differentiation, but enhanced adipogenic differentiation of human adipose tissue-derived stem cells (Yang et al. 2013; Zhu et al. 2018). In addition, TAZ induced myogenic differentiation through interaction with MyoD and activation of myogenic gene transcription (Jeong et al. 2010) (Fig. 2). YAP has also been reported to regulate osteo-adipogenic differentiation from human mesenchymal stem cells (Lorthongpanich et al. 2019) and myogenic differentiation (Watt et al. 2010; Chen et al. 2017). Although TAZ and YAP promote proliferation of myoblasts, TAZ, but not YAP, stimulates late myogenic differentiation, suggesting a distinctive role of TAZ from YAP in skeletal muscle stem cell function (Sun et al. 2017). TAZ is also essential for inducing cell proliferation and chondrocyte marker expression during chondrocyte differentiation; however, hyperactivation of YAP and TAZ inhibit chondrocyte maturation by repressing the transcription factor SOX9 (Deng et al. 2016; Goto et al. 2018). It remains to be clarified whether YAP and TAZ play redundant roles during chondrocyte differentiation and whether TAZ promotes or represses chondrocyte differentiation in vivo.

### TAZ promotes injury-induced liver regeneration and intestinal regeneration

The Hippo signaling pathway plays critical roles in liver size control and tumorigenesis through phosphorylation and activation of the large tumor suppressor kinases 1/2 (Lats1/2) and inhibition of YAP activation. Any defects in Hippo signaling molecules, including YAP, induce aberrant hepatomegaly and tumorigenesis (Dong et al. 2007; Zhou et al. 2009; Lu et al. 2010; Zhao et al. 2011). Although YAP deletion fails to restore liver mass and the expression of connective tissue growth factor (CTGF), there is no clear evidence that TAZ plays a redundant role in liver regeneration (Konishi et al. 2018; Lu et al. 2018). Recently, it was clarified that liver-specific TAZ deletion delayed liver regeneration and enhanced cell death after partial hepatectomy (Kim et al. 2019). TAZ is thus believed to stimulate liver regeneration through IL-6-mediated hepatocyte proliferation and inhibition of cell death after injury. Furthermore, TAZ is essential for intestinal regeneration following gamma-irradiation, and its overexpression is associated with intestinal tumor formation (Byun et al. 2017). Intestinal TAZ function is highly correlated with important roles in the Hippo pathway and with YAP in the control of colonic epithelial regeneration after injury (Hong et al. 2016; Yui et al. 2018). Therefore, TAZ expression as well as its activation are essential factors for tissue regeneration.

### TAZ expression is required for the maintenance of testicular structure and function

TAZ is indispensable for quantitative regulation and functional maintenance of embryonic and adult stem cells and contributes to cellular reprogramming and rejuvenation in many types of tissues. However, the functional role of TAZ is relatively lacking in reproductive organs and fertility. Jeong et al. (2017) first reported that TAZ was expressed in testicular cells and its deletion caused structural abnormality of the testicles and functional defects in fertility. TAZ deletion further facilitated testicular aging and apoptotic death of spermatogenic stem cells (Jeong et al. 2017). More recently, TAZ was found to interact with nuclear receptor 4A1 in Leydig cells and negatively modulate the expression of steroidogenic enzymes, resulting in decreased testosterone production by Leydig cells (Shin et al. 2020). In addition to the TAZ functions in the male reproductive system, TAZ has been shown to play crucial roles in oogenesis and fertilization in zebrafish and murine ovarian folliculogenesis (Dingare et al. 2018; Xia et al. 2019; Yi et al. 2019; Bernabe et al. 2020). Consistently, LATS1/2 and YAP in the Hippo pathway have been demonstrated to be essential for the proliferation of ovarian granulosa cells and maintaining normal follicle development in the female reproductive system (Plewes et al. 2019; Tsoi et al. 2019).

### TAZ modulates normal tissue homeostasis in several tissues

TAZ is expressed in most tissues and affects their specific gene expression as a mediator of the Hippo signaling pathway. TAZ deletion impairs the optic vesicle progenitor cells to form retinal pigment epithelial cells, whereas ectopic TAZ expression enhances ectopic pigmentation in optic vesicle progenitor cells (Miesfeld et al. 2015). TAZ is necessary for eye development through determination of retinal pigment epithelial cell fate. Furthermore, TAZ modulates skin homeostasis. TAZ is expressed in the nucleus of the basal layer cells of the skin and is elevated in response to wound healing. TAZ is normally localized to the cytoplasm in the dermis, but is distributed in both the nucleus and cytoplasm at 1 day after skin damage, and its deficiency markedly delays skin wound healing (Lee et al. 2014). Skin-specific TAZ deletion slows the growth of basal layer cells, leading to hair loss and inhibition of skin regeneration (Elbediwy et al. 2016a, 2016b). The association of TAZ with TEAD promotes proliferation of skin cells but the inhibition of their interaction increases the expression of kruppel-like factor 4 (KLF4), thereby increasing keratinocyte differentiation. TEAD and KLF4 regulate the activity of each other through modulation of TAZ interaction with TEAD and other transcription factors during keratinocyte differentiation (Yuan et al. 2020). Therefore, TAZ contributes in maintaining epidermal and dermal cell populations during development and also regulates skin homeostasis during wound healing. Moreover, TAZ has been suggested to promote Th17 cell differentiation and inhibit Treg cell development in immune system (Geng et al. 2017), suggesting a potential role of TAZ in autoimmune diseases. Inhibition of dysregulated TAZ expression in arthritic patient induces Treg cell-mediated anti-inflammatory effect (Du et al. 2020). TAZ also regulates lymph node differentiation, in particular, commitment and maturation of fibroblastic reticular cells (Choi et al. 2020). The fine-tuning of TAZ expression and its activity is essential for maintaining normal tissue homeostasis and limiting cancer incidence.

### Small molecules that stimulate TAZ activity are promising candidates for disease treatment

**Fig. 3:**
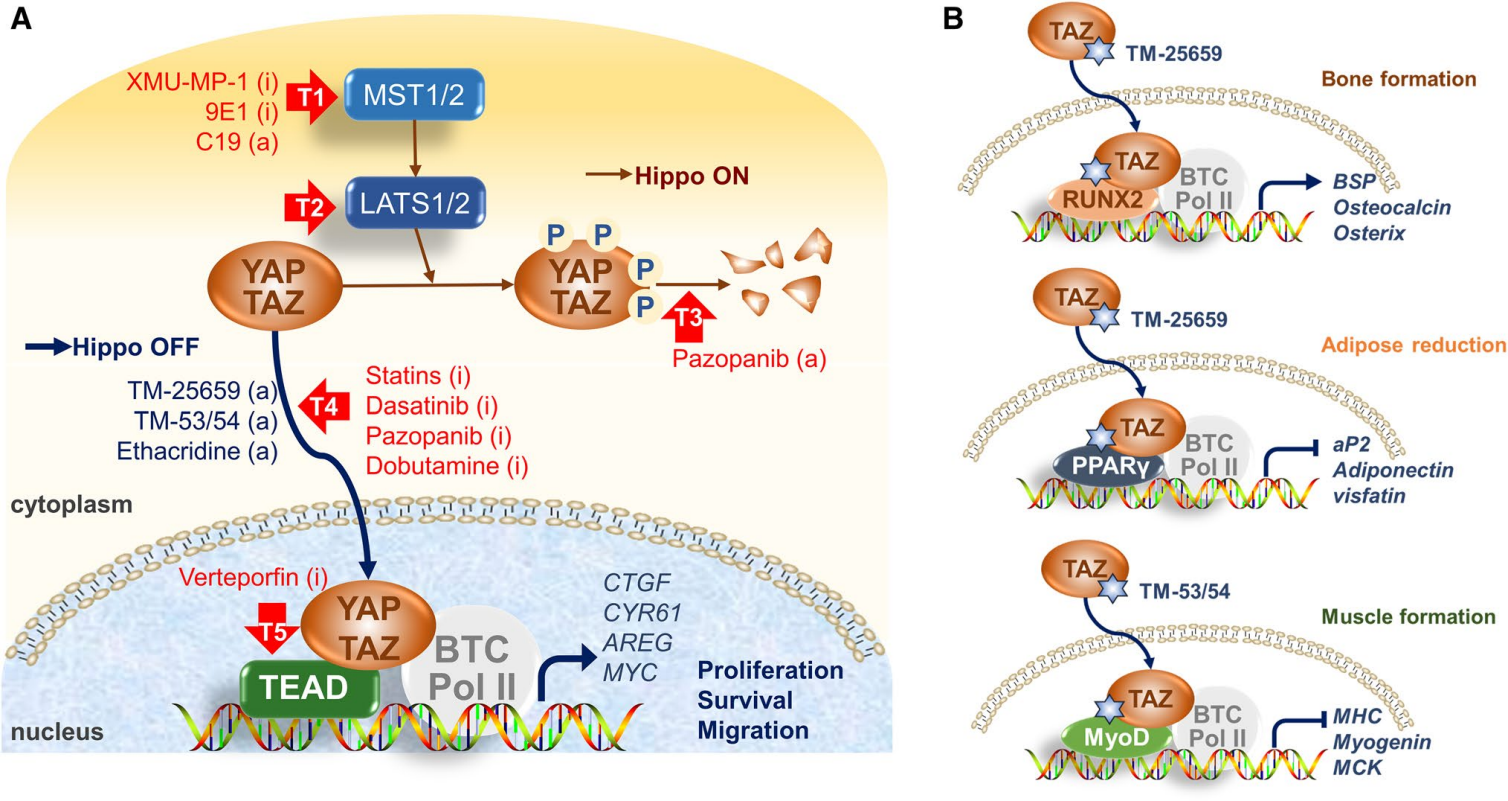
Identification of small molecules targeting the Hippo-TAZ/YAP pathway. **a** The Hippo pathway can be regulated at several target points (T1 through T5). The Hippo signaling molecules, MST1/2 (T1) and LATS1/2 (T2), are directly and indirectly affected by compounds such as XMU-MP-1, 9E1, and C19, which affect the activation of TAZ and YAP. YAP and TAZ undergo proteasomal degradation (T3), which is promoted by pazopanib. Nuclear localization of TAZ and YAP (T4) is negatively regulated by statins, dasatinib, pazopanib, and dobutamine, but positively regulated by TM-25659, TM-53/54, and ethacridine. Then YAP/TAZ-TEAD association (T5) is repressed by verteporfin. Pharmacological regulation of the Hippo-TAZ/YAP pathway affects cell proliferation, differentiation, survival, and migration. **b** Small molecules such as TM-25659 and TM-53/54 promote nuclear localization of TAZ and specifically modulate the association of TAZ with RUNX2, PPARγ, or MyoD and subsequent cell specific gene transcription for bone, adipose, and muscle, respectively

TAZ is believed to be a promising anti-cancer target because it promotes cancer cell proliferation, survival, and drug resistance. Various kinds of small molecules targeting TAZ, such as those that block the nuclear localization of TAZ, inhibit TAZ-TEAD complex formation, and suppress TAZ-TEAD target genes, are being discovered as anticancer agents. Since MST1/2 and LATS1/2 are crucial for the regulation of TAZ phosphorylation and activation, small molecules that regulate their activities in biological functions have been identified. Compound 9E1, XMU-MP-1, and neratinib inhibit MST1/2 and thus suppress the activation of LATS1/2 and subsequent YAP/TAZ-mediated cellular proliferation and tissue regeneration (Anand et al. 2009; Fan et al. 2016; Ardestani et al. 2019a). However, C19 stimulates MST-induced LATS1/2 phosphorylation and YAP/TAZ inactivation, suggesting an anti-tumor potential (Basu et al. 2014). Dobutamine has been identified as an inhibitor of YAP-mediated gene transcription (Bao et al. 2011). Dasatinib, statins, and pazopanib are known to inhibit the nuclear localization of TAZ and decrease cell proliferation and chemoresistance of breast cancer cells (Oku et al. 2015). In addition, digitoxin, verteporfin, and flufenamic acid inhibit YAP-TEAD interaction and suppress cancer growth (Liu-Chittenden et al. 2012; Sudol et al. 2012; Pobbati et al. 2015; Pobbati and Hong 2020). Although YAP inhibition is beneficial for treating many cancers, YAP activation through the inhibition of Hippo signaling is also required for pancreatic β-cell regeneration (Ardestani et al. 2019a, 2019b). Furthermore, TAZ activators are also helpful for treating diseases, including osteoporosis, diabetes, and muscular atrophy. A few compounds have been identified to enhance the nuclear localization of TAZ. Ethacridine enhances nuclear retention of TAZ and inhibits adipogenic gene expression (Kawano et al. 2015). The TAZ modulator, TM-25659, stimulates TAZ-RUNX2 interaction and the subsequent osteogenic differentiation and suppresses adipogenic differentiation by potentiating TAZ-PPARγ complex formation (Jang et al. 2012; Zhu et al. 2018). TM-25659 protects against obesity and diabetic hyperglycemia induced by high-fat diet in an animal model (Jung et al. 2015). TM-53 and TM-54 increased nuclear localization of TAZ and potentiated MyoD-induced myoblast and muscle differentiation (Park et al. 2014) (Fig. 3). These findings suggest that TAZ activators have beneficial effects in the treatment of metabolic and musculoskeletal disorders.

## Conclusions and future perspectives

Since TAZ contributes to the self-renewal and potency of stem cells associated with the acquisition of many cancer traits, targeting TAZ is considered to be more efficient in the control of several types of cancers. It is noteworthy that the development of anticancer drugs targeting TAZ is promising for treating certain types of cancers, including skin, breast, and lung cancer (Pobbati and Hong 2020). However, TAZ intrinsically serves as a physiological regulator for organ development, size control, and tissue repair and regeneration. TAZ positively or negatively regulates gene transcription through interaction with many transcription factors and modulates lineage commitment during homeostasis of many types of tissues, including the musculoskeletal system, adipose tissue, liver, digestive system, reproductive system, and skin. It seems that pharmacological activation of TAZ is required for restoring normal tissue homeostasis by increasing stem cell activity and cell lineage commitment. Considering the complex function of TAZ in cancer cells and normal tissues, including stem cells, while complete inhibition of TAZ may be effective in the treatment of cancer, there is a risk of disrupting normal tissues homeostasis. Therefore, pharmacological development of TAZ inhibitors for cancer treatment deserves a great deal of attention with respect to prediction of toxicity and side effects on tissue homeostasis. It is indispensable to develop selective inhibitors or activators that target specific binding sites between TAZ and its interacting partner, which may not interfere with normal tissue homeostasis and may contribute to desirable therapeutic effects (Pobbati and Rubin 2020).
